# Aberrant Single Exon Skipping is not Altered by Age in Exons of *NF1, RABAC1, AATF* or *PCGF2* in Human Blood Cells and Fibroblasts

**DOI:** 10.3390/genes2030562

**Published:** 2011-08-02

**Authors:** Kevin Mellert, Michael Uhl, Josef Högel, Markus Lamla, Ralf Kemkemer, Dieter Kaufmann

**Affiliations:** 1 Institute of Human Genetics, University of Ulm, Albert Einstein Allee 11, Ulm D 89070, Germany; E-Mails: kevin.mellert@uni-ulm.de (K.M.); uhl.michael@gmx.de (M.U.); josef.hoegel@uni-ulm.de (J.H.); 2 Research Group Chemical Function in Biosystems, University of Ulm, Albert Einstein Allee 11, Ulm D 89070, Germany; E-Mail: markus.lamla@uni-ulm.de; 3 Max Planck Institute for Intelligent Systems, Stuttgart, ZWE Biomaterials, Heisenbergstrasse 3, Stuttgart 70569, Germany; E-Mail: ralf.kemkemer@mf.mpg.de

**Keywords:** splicing, noise, splice errors, mRNA processing

## Abstract

In human pre-mRNA splicing, infrequent errors occur resulting in erroneous splice products as shown in a genome-wide approach. One characteristic subgroup consists of products lacking one cassette exon. The noise in the splicing process, represented by those misspliced products, can be increased by cold shock treatment or by inhibiting the nonsense mediated decay. Here, we investigated whether the splicing noise frequency increases with age *in vivo* in peripheral bloods cells or *in vitro* in cultured and aged fibroblasts from healthy donors. Splicing noise frequency was measured for four erroneously skipped *NF1* exons and one exon of *RABAC1, AATF* and *PCGF2* by RT-qPCR. Measurements were validated in cultured fibroblasts treated with cold shock or puromycin. Intragenic but not interpersonal differences were detected in splicing noise frequencies *in vivo* in peripheral blood cells of 11 healthy donors (15 y–85 y) and in *in vitro* senescent fibroblasts from three further donors. No correlation to the age of the donors was found in the splicing noise frequencies. Our data demonstrates that splicing error frequencies are not altered by age in peripheral blood cells or *in vitro* aged fibroblasts in the tested exons of the four investigated genes, indicating a high importance of correct splicing in these proliferating aged cells.

## 1. Introduction

Cotranscriptional splicing of human pre-mRNA involves the interaction of several distinct protein factors and 5 snRNAs with sequences specific to the pre-mRNA [1]. The U1 and U2 snRNAs comprise the U1 respective U2 snRNP. U1 binds to the 5' splice site, the U2 snRNP complexes are directed to the branch point by U2AF which binds to the polypyrimidin tract. Further spliceosomal compounds (U4, U5, U6) join together with the RNA-bound complexes and SR proteins to build the spliceosome. Two transesterification reactions lead to the release of the intron and to the ligation of the exons [2]. Errors resulting for example in transcripts lacking one or more cassette exons rarely occur in splicing [3]. Such erroneous transcripts were first observed in low temperature (cold shock) treated peripheral blood cells whilst screening for *NF1* mutations at RNA level [4,5]. In a systemic approach using real-time quantitative PCR (RT-qPCR), these transcripts were found in all tested *NF1* exons at levels varying between 0.007 and about 2% of the total amount of constitutive spliced (wildtype) *NF1* transcripts in collected tissues [6]. Erroneous transcripts were also found in the tumor suppressor genes *NF2* and *TSC2* both in cultured cells and tissues. The levels of these erroneous transcripts increased in cells cultured in media with a low pH or at high temperature, conditions found in tumor tissue [7]. In addition, multi-exon skipping was found at very low frequencies in *NF1, TSC2, RPL23* and *UBA52* in cultured human cells [1,6,7].

It is debated whether these erroneous transcripts (splicing noise) are caused by a stochastic missing exon recognition, insufficient fidelity of transcription, inaccuracy of the splicing machinery or somatic mutations in single cells in splice regulating sequences or genes such as *SC2* [1,6-8]. In alternative splicing, the recognition of exon splice sites depends on splice site strength, intron length and a sufficient concentration of splice-relevant proteins such as SC2 [8]. SC2 and SMN (a protein whose expression is decreased in spinal muscle atrophy (SMA)) also influence the splicing noise frequencies as shown in investigations of *RPL23* and *UBA52* [1]. In a recent genome-wide approach, it was shown that the amount of splicing errors can be correlated to the expression rate of genes [9].

There are several RNA surveillance mechanisms degrading misspliced mRNAs [10]. One of them, the nonsense-mediated mRNA decay (NMD), degrades mRNA isoforms containing premature termination codons [11]. Inhibition of NMD by puromycin treatment or *hUpf1* knockdown increases splicing noise frequencies [1,12].

We are interested in age-dependent changes in splicing noise frequencies in human cells. There are several models which explain the complex phenotype of aging [13,14]. One of these models centers on the age-dependent increase of stochastic mutations in nuclear and mitochondrial DNA. Until now, age-dependent effects on gene transcription have not been investigated with the same intensity as alterations in DNA structure. However, there is data suggesting an age-dependent expression pattern of genes [15]. One of the most interesting findings in this field is the observation that cell-to-cell variation in gene expression (transcriptional noise) is increased in aged cells in isolated single cells [16]. Additionally age-related changes in alternative splice site usage have been described in specific genes [2], while age-dependent alterations in splicing noise frequencies, represented by cassette ex on skipping, have not yet been investigated.

A highly reliable method of measuring splicing noise frequencies is the relative quantification of an erroneous product compared to the wildtype product by RT-qPCR [6]. Because this method is very sensitive, it is a prerequisite to avoid artefacts such as those incurred by incorrect RNA isolation or mispriming. The detection method can be validated by the measurement of exon skips in cultured fibroblasts treated with cold shock or puromycin, conditions known to increase splicing noise frequencies. To test whether splicing errors are correlated to the transcription rate or if they are caused by inaccurate exon recognition, reduced transcription fidelity or mutations in splice genes in single cells [1,6,17], we investigated intragenic and interpersonal variations in splicing noise frequencies in several exons of one single gene. This gene, the tumour suppressor *NF1*, contains at least 58 exons and has been extensively investigated for alternative splice products, consequences of germline mutations on splicing and splicing noise [6,7,18]. Additionally one exon skip was measured in each of three other genes, the housekeeping genes *PCGF2, RABAC1* and *AATF*.

*In vivo*, the most accessible human tissue for these investigations is represented by carefully isolated peripheral blood cells. Using these cells, we showed an unchanged high fidelity in splicing in aged peripheral blood cells in the investigated exons of the selected genes *NF1, PCGF2, RABAC1* and *AATF*. *In vitro* investigations of old fibroblasts revealed comparable results.

## 2. Results and Discussion

### 2.1. Results

#### 2.1.1. Reliable Detection of Splicing Noise Frequencies by RT-qPCR

**Figure 1:**
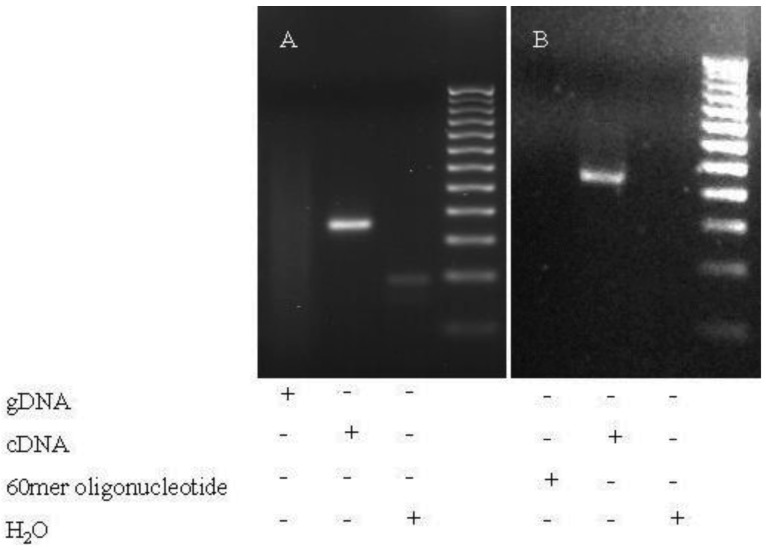
(**a**) The specificity of NF1-Δ46 skip primers. (**b**) The specificity of RABAC1-Δ4 *s*kip primers: The specificity of the primers to detect NF1-Δ46 or RABAC1-Δ4 erroneous splice products was tested on genomic DNA, cDNA of fibroblasts of a young donor (FP1) and 60mer oligonucleotides representing the wildtype sequence in standard PCR. The primer pairs did not generate products on genomic DNA and oligonucleotides after 60 cycles of amplification implicating that no products can be amplified as a reason of mispriming at sequences that match partly with the target sequence. To detect the length of the resulting products in the 3% agarose gel, a 25 bp marker was used. In lane 3 of panel A (water control) a weak primer band is visible.

Splicing noise frequencies were measured by RT-qPCR of the regular *NF1, PCGF2, RABAC1* and *AATF* transcripts (wildtype products) in relation to the products without the skipped exons (NF1-Δ38, NF1-Δ39, NF1-Δ46, NF1-Δ52, PCGF2-Δ10, RABAC1-Δ4 and AATF-Δ3). The investigated skipped exons differ in length, in upstream and downstream intron length, splice site strengths (calculated with SplicePort [19]) and in generating a frameshift as consequence of the exon skip (Table 1). The specificity of the skip primers was tested by PCR (60 cycles) on genomic DNA and wildtype 60mer oligonucleotides. The oligonucleotides represent the wildtype sequence at the position of skip primer binding and enable detection of mispriming of the skip primers (Scheme I). The method was tested using published *UBA52* and *RPL23* primers known to misanneal at low frequency [1]. PCRs using those primers resulted in clear bands on gDNA. In contrast, in experiments using the skip primers used in our measurements, no products were found in PCR on gDNA or oligonucleotides, whereas PCRs on cDNA of fibroblasts resulted in the expected products (Figure 1). This implicates that misannealing with these primers may occur at even lower frequency than that published for the *UBA52* and *RPL23* primers (found to misanneal <1:1000).

**Table 1:** Structural data to the investigated exons: Length (bp) of the *NF1, PCGF2, RABAC1* and *AATF*-exons, upstream introns, downstream introns, splice site strengths of the donor and acceptor sites (score calculated with SplicePort) and the classification as in-frame (i. f.) or out of frame (o. f.) exons.

	**Exon**	**Intron**		**Splice site strength**		

		**Upstream**	**Downstream**	**Donor**	**Acceptor**	**Classification**
*NF1* Exon 38	341	1246	2456	0.99	−0.28	o. f.
*NF1* Exon 39	203	2456	4339	1.50	1.39	o. f.
*NF1* Exon 46	102	564	1699	0.53	−0.04	i. f.
*NF1* Exon 52	123	4045	377	0.11	0.93	i. f.
*PCGF2* Exon 10	81	2182	489	1.72	2.21	i. f.
*RABAC1* Exon 4	102	1166	83	0.42	0.72	i. f.
*AATF* Exon 3	411	2480	473	1.34	2.16	i. f.

**Scheme I:**
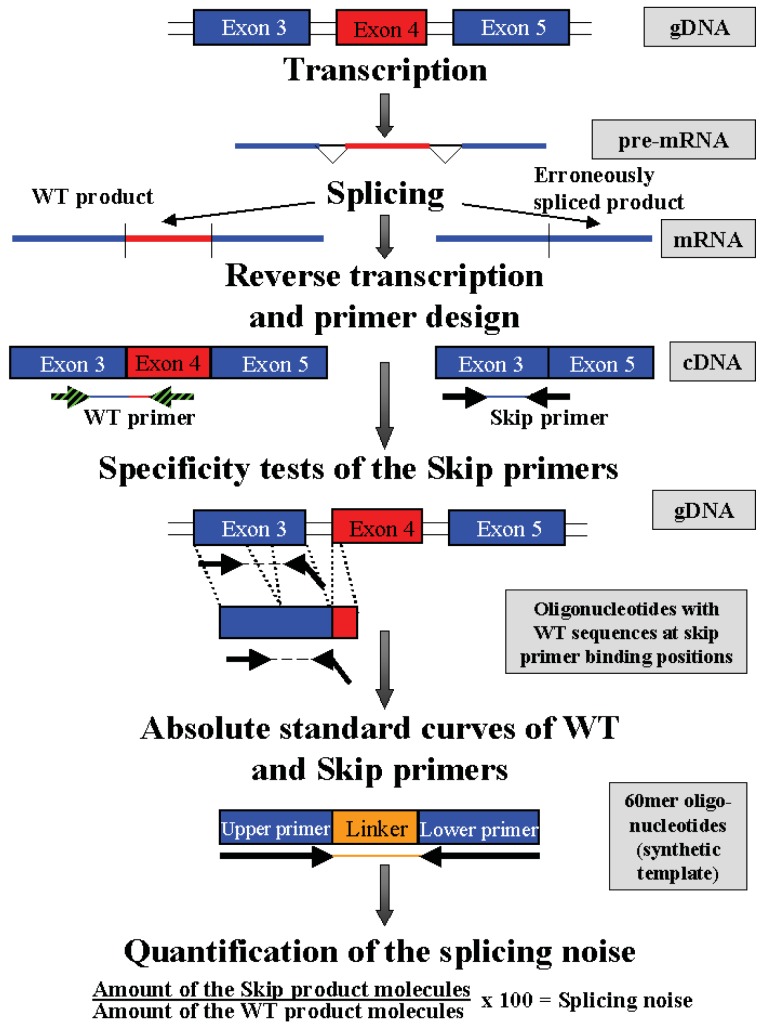
General strategy of the PCR approach using the example of RABAC1-Δ4: After transcription of the gene, mRNA splicing leads either to a wildtype (WT) or an erroneously spliced product. The whole RNA was reverse transcribed to cDNA. The primers to detect the wildtype product are positioned in two consecutive exons (exon 3 and 4). The determining primers to detect the erroneously spliced product were designed as exon-boundary primer ranging over the speculative new exon-exon boundary after exon skipping (exon 3 and 5). To test the specificity of the skip primer pairs, gDNA and oligonucleotides representing the WT sequence at the skip primer binding position were used as template in a 60 cycle PCR. Determining skip primers can bind to the gDNA, oligonucleotide or wildtype cDNA only with 3–7 bases, so no product is amplified. Amplifications with cDNA as a template resulted in the clear products of the expected length. Absolute standard curves were performed by adding known amounts of synthetic templates representing the WT and skip primer sequences with a linker sequence in between. The amount of wildtype product molecules and skip product molecules in the measured cDNAs were calculated using the absolute standard curves.

**Figure 2:**
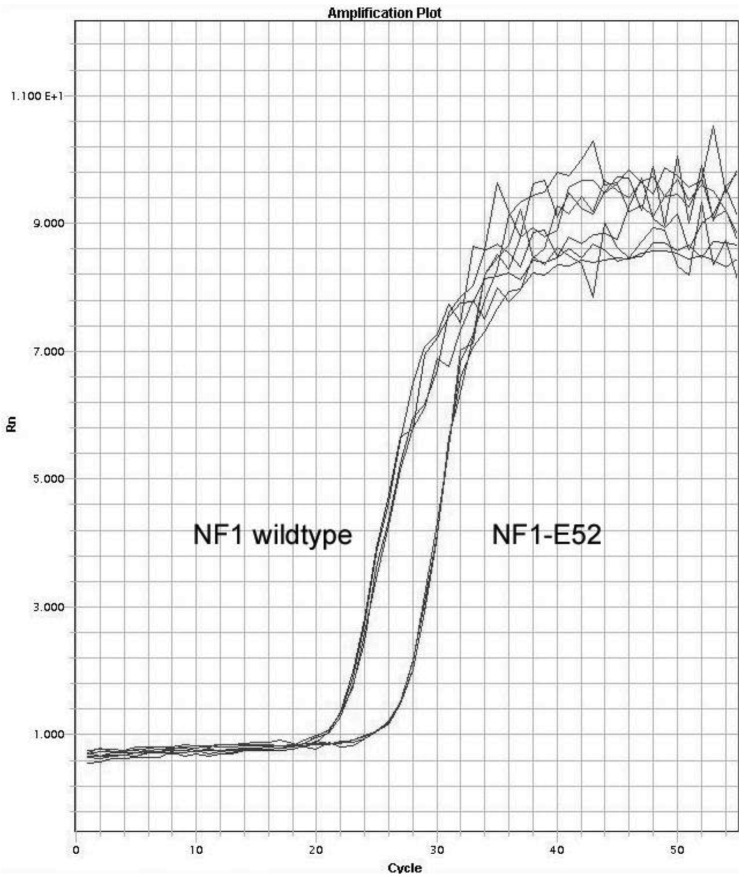
qPCR to detect the *NF1* wildtype and the transcript NF1-Δ52: The quantification of *NF1* wildtype and of the misspliced transcripts in the cDNA of fibroblasts was performed by qPCR. Original amplification curves are represented. The amounts of mRNA molecules were calculated using an absolute standard curve. Splicing noise = the percentage of skip product in relation to the wildtype products. qPCR measurements were performed fourfold.

In *NF1*, the amount of the regular *NF1* product was measured by qPCR using three different primer pairs binding to sequences flanking the skipped exons. The differences in the measurements of *NF1* expression ranged within the deviation of multiple measurements expected from a single primer pair (Table 2). Therefore, in the following qPCR experiments, regular products were detected by using the WT38-39 primers only. Measurement of the four misspliced *NF1* products resulted in low SDs (Figure 2), but the respective CT values differed in the cDNA of peripheral blood cells (Table 3) indicating that not every exon is erroneously spliced at the same frequency.

**Table 2:** CT values of RT-qPCR of the wildtype *NF1* products: Different *NF1* primers were used in cDNA of fibroblasts of FP1 (mean, n = 3, ±SD).

**Wildtype primer pair**	**CT value**
NF1WT38-39	23.78 ± 0.05
NF1WT45-46	23.66 ± 0.02
NF1WT52-53	23.74 ± 0.10
Mean	23.73 ± 0.06

**Table 3:** CT values, calculated mRNA molecule amounts and relative percentage of the skip products measured in RT-qPCR: CT values of RT-qPCR, the calculated mRNA amount and the corresponding relative amounts of the regular *NF1* product (NF1WT38-39) and the *NF1* products missing exon 38, 39, 46 or 52 (NF1-Δ38, NF1-Δ39, NF1-Δ46, NF1-Δ52) in cDNA (100 ng RNA equivalent) of peripheral blood cells (BP1, mean, n = 4, ±SD).

**Product**	**CT value**	**Calculated mRNAs**	**Relative amount (%)**
NF1WT38-39	21.69 ± 0.07	9330	
NF-Δ38	32.92 ± 0.47	11	0.11
NF-Δ39	27.81 ± 0.13	296	3.17
NF1-Δ46	33.05 ± 0.14	10	0.11
NF1-Δ52	26.6 ± 0.10	634	6.79

The reliability of splicing noise frequency detection was also investigated in fibroblasts treated by cold shock or puromycin. After treatment with cold shock, a clear increase of splicing errors was detected in three of four investigated *NF1* exons and in both *PCGF2* and *RABAC1* (Table 4). Treating the fibroblasts with puromycin leads to an increase in splice errors in both 3 of the 4 *NF1* exons and in the investigated exons of *RABAC1* and *AATF*. These results indicate that the RT-qPCR method is able to detect differences in splicing noise frequency of the *NF1* exons. Testing dilutions of cDNAs revealed, that the detection limit of this method is a decrease of about 40% between two samples (data not shown).

**Table 4:** Amount of erroneously spliced *NF1, PCGF2, RABAC1* and *AATF* products: Relative amount of erroneously spliced *NF1* (NF1-Δ38, NF1-Δ39, NF1-Δ46, NF1-Δ52), *PCGF2* (PCGF2-Δ10), *RABAC1* (RABAC-Δ4) and *AATF* (AATF-Δ3) products in relation to the wildtype products (%, mean, n = 4 (measurements in RT-qPCR)) in cultured fibroblasts untreated or treated by cold shock or puromycin.

	**NF1-Δ38**	**NF1-Δ39**	**NF1-Δ46**	**NF1-Δ52**	**PCGF2-Δ10**	**RABAC1-Δ4**	**AATF-Δ3**
untreated	0.03	0.11	0.06	3.26	6.7 × 10^−10^	1.61	4.1 × 10^−9^
coldshock	0.08	0.35	0.08	5.76	1.8 × 10^−9^	5.70	n.d.
puromycin	0.13	0.47	0.07	6.59	9.7 × 10^−10^	4.15	9.4 × 10^−9^

No significant correlation of the splicing noise frequency to the exon-specific parameters exon or intron length or the splice site strength could be found in this small sample of exons.

#### 2.1.2. NF1 Splicing Noise Frequencies in Aged Cultured Fibroblasts

**Figure 3:**
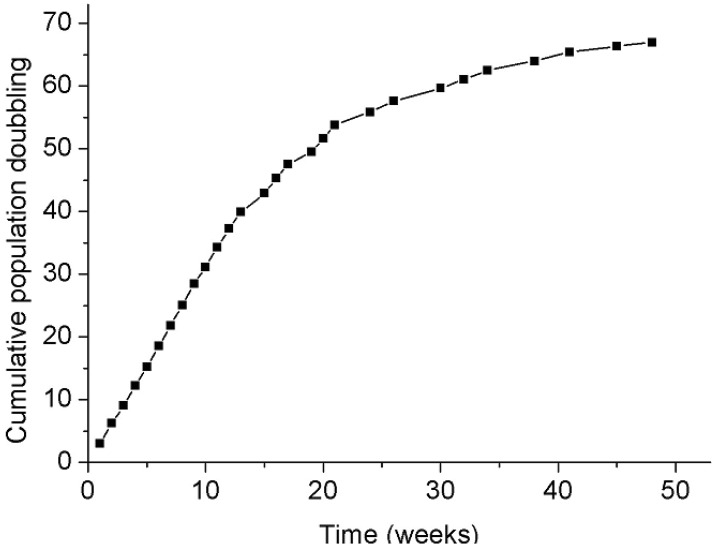
Cumulative population doublings of human fibroblasts of a 3 year old healthy donor (FP1): Cell doublings were estimated at the indicated times by counting the living cells. Fibroblasts of FP1 of passages 4 and 27, representing 4 or 45 weeks of cell culture, were investigated for splicing noise. Cells of passage 4 were defined as young, cells of passage 27 as old.

Splicing fidelity may be influenced *in vitro* by replicative senescence. To test this, cultured fibroblasts from three donors (FP1, FP7 and FP8) were investigated at different passages (3–4 and 24–29), representing respectively 3–4 and more than 40 weeks of culture. The replicative senescence was shown by reduced population doubling (Figure 3), the altered morphology of the cells and the determination of the telomere length. A significant decrease in splicing noise frequency (p < 0.05; paired t-test) could be observed in *NF1* exon 39 in *in vitro* aged fibroblasts of all three donors (FP1, FP7, FP8), but overall no significant change in splicing error frequency correlating with replicative senescence could be found (p = 0.39; sign test of the raw data; Table 5A). In addition, cultured fibroblasts (passages 5–6) from four donors differing in age (3–67 years) were investigated by pairwise testing a culture of a young and elderly donor (Table 5B). A comparison on the basis of four exons in two elderly and two young donors revealed slightly lower noise for the latter. This may however be attributable to chance (p = 0.52, ANOVA) and hence, no correlation between splicing noise frequency and the age of the donors could be detected under our culture conditions *in vitro*. The mean values ±SD per tested fibroblast pair over all tested *NF1* exons point out that there is no significant difference between young and old fibroblasts/donors in our collective regarding the splicing error frequencies.

**Table 5:** Differences in the amount of erroneous splice products *NF1-Δ38 NF1-Δ39, NF1-Δ46, NF1-Δ52* between cultured young (normalized to 1.00) and old fibroblasts: [A] Effect of *in vitro* aging: cDNA of cultured fibroblasts from three donors (FP1, FP8 and FP7, aged 3, 8 and 22.5 years) in the passages 3–4 (young fibroblasts) and 24–29 (old fibroblasts) was investigated for the relative amount of erroneous *NF1* products. The age of the donor, source of the obtained fibroblasts, amount of erroneously spliced products in cDNA of old fibroblasts normalized each to the young fibroblasts of *NF1* exons 38, 39, 46 and 52 and the mean over all tested exons per test pair (± standard deviation) are given. Paired comparison of low with corresponding high passage fibroblasts of the same donor yields no evidence of significant difference in splicing error frequencies (p = 0.39; sign test). [B] Effect of the age of the donors of fibroblasts: pairwise tested cDNA of cultured fibroblasts of young and old donors; pair 1: FP3 (24 years)–FP4 (67 years), fibroblasts obtained from the prepuce; pair 2: FP2 (3 years)–FP5 (65 years), fibroblasts obtained from the upper arm. The amount of erroneously spliced products found in fibroblasts of the old donors were normalized to the respective young donor fibroblast amounts. No significant difference of noise frequencies could be detected between fibroblasts of young and elderly donors (p = 0.52; ANOVA).

**A.**							

**Donor**	**Passages**	**Source**	**NF1-Δ38**	**NF1-Δ39**	**NF1-Δ46**	**NF1-Δ52**	**Mean**
FP1	4/27	Prepuce	1.91	0.63	0.48	1.22	1.06 ± 0.65
FP7	3/24	Prepuce	0.59	0.28	1.16	0.97	0.75 ± 0.39
FP8	3/29	Prepuce	0.89	0.43	0.53	1.14	0.75 ± 0.33

**B.**							

Pair	**Donor age**	**Source**	**NF1-Δ38**	**NF1-Δ39**	**NF1-Δ46**	**NF1-Δ52**	**Mean**
FP3/FP4	24/67	Prepuce	0.98	0.42	0.84	0.70	0.74 ± 0.24
FP2/FP5	3/65	Upper arm	1.43	0.61	1.34	2.84	1.56 ± 0.93

#### 2.1.3. Intragenic, but not Age-Dependent Interpersonal Variation in NF1 Splicing Noise Frequencies in Peripheral Blood Cells

In order to investigate splicing noise frequencies *in vivo*, carefully and rapidly isolated RNA from peripheral blood cells of 11 obviously healthy Caucasian male donors of different ages was tested. The splicing noise frequencies differed substantially between the four tested *NF1* exons indicating an intragenic variation in splicing (Table 6). This difference did not depend on the exon classification in-frame or out of frame. Splicing fidelity can be altered by germline mutations in genes of the splicing machinery [20,21] and may also vary interpersonally. There was no evidence that the *NF1* splicing noise frequencies are different between young (15–23 years, n = 7) and old donors (61–85 years, n = 4; Table 6). Interpersonal differences regarding the specific exons exist (up to 3.3 fold between the lowest and highest error frequency in NF1-Δ46) but seem to be independent from the age of the donor. Furthermore, there are no donors with exclusively high or low splicing error frequencies in our collective.

**Table 6:** Relative Amount of misspliced *NF1, RABAC1* and *AATF* products in peripheral blood cells: Relative amount of erroneously spliced *NF1, RABAC1* and *AATF* products in relation to the wildtype products (%, mean, SD, n = 4) in isolated peripheral blood cells of healthy young (15–23 years) and elderly donors (61–85 years) as detected by RT-qPCR. No significant differences between the age groups (p = 0.33; ANOVA, AATF-Δ3 omitted) could be found. P-values of exon-wise comparison of the age groups are given in the last row of the table (two sample t-test).

**Donor**	**Age**	**NF1-Δ38**	**NF1-Δ39**	**NF1-Δ46**	**NF1-52**	**RABAC1-Δ4**	**AATF-Δ3**
BP11	15	0.11	2.33	0.09	7.49	3.43	2.6 × 10^−9^
BP10	16	0.08	3.68	0.09	8.77	3.17	2.0 × 10^−9^
BP9	16	0.08	2.23	0.03	6.28	5.55	6.7 × 10^−10^
BP8	17	0.11	2.74	0.10	6.04	4.38	n.d.
BP7	17	0.08	5.35	0.09	9.69	1.91	n.d.
BP6	19	0.10	3.79	0.08	6.89	3.14	1.5 × 10^−9^
BP5	23	0.07	3.78	0.06	9.60	1.99	2.7 × 10^−10^
Mean		0.09	3.42	0.07	7.82	3.37	3.1 × 10^−9^
SD		±0.02	±1.09	±0.02	±1.53	±1.29	±2.9 × 10^−9^
BP4	61	0.09	4.59	0.07	8.15	4.60	1.6 × 10^−9^
BP3	66	0.11	2.76	0.07	8.90	2.65	2.5 × 10^−10^
BP2	77	0.07	4.07	0.06	7.38	3.03	1.1 × 10^−9^
BP1	85	0.11	3.17	0.11	6.79	4.07	7.6 × 10^−10^
Mean		0.10	3.65	0.08	7.81	3.59	9.4 × 10^−10^
SD		±0.02	±0.83	±0.02	±0.92	±0.90	±5.8 × 10^−10^
p-values		0.66	0.72	0.98	0.98	0.77	0.43

#### 2.1.4. PCGF2, RABAC1 and AATF Splicing Noise Frequencies in Peripheral Blood Cells

Three housekeeping genes were also investigated for exon skipping. In *PCGF2* in the peripheral blood cells of seven young and four elderly donors the skip product PCGF2-Δ10 was not detectable, whereas in the cultured fibroblasts of a young donor (FP6) it could be found at a level of 6.7 × 10^−10^% and was increased by cold shock to 1.8 × 10^−9^%. In *RABAC1* and *AATF* the amounts of the skip products were detectable in cultured fibroblasts and could be increased by cold shock or puromycin treatment (Table 4). In peripheral blood cells of the 11 donors the amount of *RABAC1* and *AATF* erroneously spliced exons varied, but did not seem to be systematically different between young and elderly people (Table 6).

### 2.2. Discussion

Measurements of splicing noise frequencies and investigations into their cellular and organic consequences are in their infancy [22]. To investigate the interpersonal and age-dependent variation in splicing error frequencies, peripheral blood cells from healthy donors were investigated for exon skipping in *NF1, PCGF2, RABAC1* and *AATF* by RT-qPCR.

There are several pitfalls in the measurement of the very rare products from splicing noise. One is mispriming in qPCR. To solve this problem, all primers were tested on specific oligonucleotides and/or gDNA for mispriming. The second pitfall is an unreliable RNA isolation. Specific protocols were used to avoid disturbances by cold shock induced splicing noise especially in peripheral blood cells. Third are disturbances by variable effects from the RNA surveillance mechanisms. In general, mRNAs with skipped in-frame exons are not a target for the NMD which eliminates targets with premature termination codons [11]. Therefore, in this study the majority of the selected skipped exons are in-frame. However, the inhibition of NMD by puromycin in cultured fibroblasts resulted in increased splicing noise in three of five in-frame exon skips and both tested out of frame exons. In-frame exon skips should not be altered by puromycin treatment for that the NMD should not degrade mRNAs lacking an in-frame exon. The increase of mRNAs with in-frame skips may be due to effects of puromycin treatment independent from NMD inhibition.

In peripheral blood cells, the erroneous exon skipping frequency was found to be 2.49% on average, varying between 1.2 × 10^−9^ (AATF-Δ3) and 7.81% (NF-Δ52). his range fits to that found in EST libraries, where irregular splicing products are suggested to vary between 1 and 10% of all cDNAs [23]. Using deep RNA sequencing, Pickrell *et al.* showed that exons have an average splicing error frequency of approximately 0.7% [9]. This average error percentage is lower than those found in our measurements in peripheral blood cells. This may be due to the small collective of the investigated exons in our study.

Whereas fractions of misspliced products of less than 1% of the wildtype product can be more easily defined as errors, fractions with levels greater than 2% implicate that they might have a dedicated function. The NF1-Δ52 isoform was suggested to be functional [6] but to our knowledge the other investigated exon skips have not yet been described as functional isoforms. In our survey, intragenic and intergenic variations in splicing noise were found in the peripheral blood cells of all 11 donors, but the interpersonal variation was, although being partial higher than expected (up to 3.3 fold between individuals), independent from the age of the donors. The patterns of single donor splicing error frequencies within the whole collective show that there are no exclusively high or low frequencies regarding single donors. This indicates that there are no donors that have a more accurate splicing machinery than others in our collective. The intragenic differences were found not to be related to the exon classification in-frame or out of frame. In particular, no correlation to the age of the donors could be found. Other variants of splicing noise such as multiple exon skipping or alternative splice site usage are said to be altered by aging [2]. Considering this, the consistency of splicing noise frequency based on cassette exon skipping is surprising and indicates that not all types of splicing noise are age-dependent.

Several explanations for the missing alteration with age in cassette exon skipping in these cells can be considered: (1) splicing noise frequencies are not influenced by the aging process; (2) the investigated proliferating peripheral blood cells are selected *in vivo* for reduced splicing noise frequencies; (3) the RNA surveillance mechanisms are more active in aged peripheral blood cells; or (4) there are age-dependent differences in splicing noise frequencies, but the alteration is too small to be detected by our method. In this case, changes in the noise frequencies would be below our detection limit and would therefore be smaller than about 40%. It was shown that there is an approximately 2-fold splicing error increase in the neurodegenerative disorder spinal muscular atrophy (SMA) [1]. Changes in this range would clearly be detected by our method. Nevertheless, additional experiments are necessary to clarify the significance of splicing noise in aged cells, especially in non-proliferating tissue (e.g., in mice) and cells with knocked down surveillance mechanism genes.

There are several hypotheses as to the reasons for splicing noise. In one, the amount of erroneous splice products is linked directly to the amount of functional products [23]. In *NF1*, the splicing noise frequencies varied in the different exons, indicating that other variables influence the frequency of splicing noise besides the transcription rate. The observed pattern suggests that *NF1* splicing noise frequencies are not correlated to the fidelity of transcription, mutations in splicing regulating sequences or genes in single cells. A crucial factor for the different noise frequencies in different exons may be the strength of the splice sites. This seems to be reasonable since a missing splice acceptor site recognition would lead to the usage of the next possible acceptor site and therefore to a missing exon recognition. In our survey, the splicing noise frequencies do not correlate with the splice site strengths. Nevertheless, the exons with the two highest upstream acceptor splice site strengths showed the lowest skipping frequencies. Other factors may also possibly influence the splicing noise frequency of an exon.

Aside from the sequence of a gene, other cellular variables influence splicing noise frequencies. This is shown by the differences in *NF1* splicing noise frequencies between cultured fibroblasts and peripheral blood cells found in this study or between different tissues as reported by others [6]. Additionally, increased splicing noise may be related to symptoms in diseases such as SMA caused by a mutation in splicing regulating genes [20].

## 3. Experimental

### 3.1. Culture of Fibroblasts and Obtaining of Blood Samples

The processing of tissue and the preparation of the human fibroblasts was performed as described [7]. Biopsies from eight obviously healthy Caucasian male donors from southern Germany (FP1-FP8) were obtained from the prepuce or the skin of the upper arm. The research carried out was in compliance with the Helsinki Declaration (Ethikkommission Universität Ulm, A 185/09). The fibroblasts were cultured in Dulbecco's modified eagle medium (DMEM) with 10% fetal bovine serum (FBS) at 37 °C and 5% CO_2_ in 60 cm^2^ dishes (3.0 × 10^6^ cells) for 3 days. Cumulative population doublings were investigated in fibroblasts of three donors. For cold shock, fibroblasts were cultured additionally for 24 h at 20 °C [7]. To inhibit the NMD, cells were treated by 100 μg puromycin/ml for 12 h [1].

To isolate RNA of peripheral blood cells, 10 ml blood samples were taken from 11 obviously healthy Caucasian male donors from southern Germany of different ages (15–85 years). All donors participating in this study gave informed consent prior to the blood isolation.

Telomere length was determined using telomere primers as described [24]. PCRs were performed (95 °C for 15 seconds, 54 °C for 2 minutes, 25 cycles) and amplified products were run on a 3% agarose gel. Fibroblasts of lower passages showed longer maximal amplicons than fibroblasts of high passages.

### 3.2. Isolation of RNA of Cultured Fibroblasts and Peripheral Blood Cells

To isolate RNA, cultured fibroblasts were lysed directly on ice on the dishes by covering with lysis buffer (RNeasy Mini Kit, Qiagen, Hilden, Germany) containing 1% mercaptoethanol for 2 minutes, harvested with a cell scraper and disintegrated using a Qiashredder column. The following RNA isolation steps were performed according to the manufacturer's protocol (RNeasy Mini Kit, Qiagen, Hilden, Germany). Total RNA of peripheral blood cells was isolated with the LeukoLock Total RNA Isolation Kit (Ambion, Austin, USA). This method allows RNA stabilization within 10 minutes and complete isolation within less than 90 minutes, ensuring minimal fluctuations in RNA levels. The synthesis of cDNA was performed with the Superscript III kit (Invitrogen, Karlsruhe, Germany) using random hexamers.

### 3.3. Investigated Exons

The possibility of an erroneous but infrequent skipping of nearly every exon in a gene was demonstrated for *UBA52* and *RPL23* [1] and in a genome-wide approach for nearly all genes [9]. The detection of the erroneously spliced products of several *NF1* exons has already been published [6,7]. Considering the published results in this study, no obvious correlation was found between the position of tested *NF1* exons within the gene and the amount of erroneously spliced products. Therefore for this study, the *NF1* exons 38, 39, 46 and 52 were randomly selected from the list of published exons. Additionally, in-frame exons of the three housekeeping genes, *PCGF2* (exon 10), *RABAC1* (exon 4) and *AATF* (exon 3) were selected. There were three rationales for the selection of these additional exons in our study. First, these genes are expressed in most cell types, especially in cultured fibroblasts and peripheral blood cells. Second, these exons are not spliced alternatively, thus a strict discrimination between alternative and erroneous splicing can be achieved. The finding of a transcript lacking the interesting exon in the Ensembl database excludes this exon. Third, these exons are classified as in-frame exon. Skips of in-frame exons normally do not lead to premature stop codons. Therefore, emerging products lacking in-frame exons are not targets for the NMD.

### 3.4. qPCR of the Regular Transcripts and the Exon Skip Products

cDNA splicing noise frequencies were measured in *NF1* for two in-frame (exon 46 and 52) and two out of frame exons (exon 38 and 39), in *PCGF2* for the in-frame exon 10, in *RABAC1* for the in-frame exon 4 of and in *AATF* for the in-frame exon 3 by qPCR (Table 1). The nomenclature of the exons of all four genes was assumed from the Ensembl data base (http://www.ensembl.org) and may therefore differ from the nomenclatures used in other publications (Ensembl: *NF1*: E38, E39, E46, E52 match to *NF1*: E29, E30, E37, E43 in [6]). To detect erroneous splice products, exon boundary primers were used. The 3′-end of all primers overlaps the new exon-exon boundary generated by skipping the investigated exon (Supplementary Table 1, Scheme I). The primers used for the detection of splicing noise in *NF1* were established by others [6], including tests on cloned wildtype and exon-skipped products. The specificity of the skip primers was tested on genomic DNA in standard PCR (60 cycles) and in *PCGF2, RABAC1* and *AATF* using 60mer oligonucleotides including the primer binding sequences of the wildtype cDNA (Supplementary Table 2) (expected PCR products were detectable on a cDNA target after 30–45 amplification cycles). The erroneously spliced and the corresponding regular products were amplified in qPCR using the QuantiTect SybrGreen kit (Qiagen, Hilden, Germany) in a 7900 HT Fast Real-time PCR-System (Applied Biosystems, Foster City, USA). The splicing noise frequencies were calculated as relative amounts of the exon skipped products to the wildtype products. Amounts of the mRNAs in 100 ng RNA equivalent cDNA were calculated via absolute standard curves performed with known amounts of synthetic templates (60 mer oligonucleotides representing the primer sequences (WT and skip) and a linker sequence, Supplementary Table 2A). Splicing noise = Amount of the skip product molecules/Amount of the wildtype products molecules * 100%.

### 3.5. Statistical Analysis

Splicing noise frequencies and other quantitative traits were summarized using their mean value ± standard deviation (SD). Within each exon, comparisons of noise frequencies between young and elderly donors were carried out using the two-sample t-test. In case of comparison of the splicing error frequencies between different passages of the same donor, the paired t-test was used. Splicing noise levels between exons and age categories were compared simultaneously using fixed effects analysis of variance (ANOVA), hereby considering in the underlying model that noise frequencies have remarkably different levels of dispersion in different exons. Associations between quantitative traits were assessed using the Pearson correlation coefficient. All statistical testing was done on a purely explorative basis without adjustment for multiple testing. Occasionally, a p-value ≤ 0.05 is termed “significant”.

### 4. Conclusions

Our study shows that splicing is a highly controlled process with a continued high fidelity in the course of aging in the investigated exons of the 4 studied genes in peripheral blood cells and cultured fibroblasts. This indicates a high importance of correct splicing in these proliferating aged cells.

## Support

This research was supported by DFG (Kaufmann, DFG KA 898/6-1).
